# Exercise combined with a probiotics treatment alters the microbiome, but moderately affects signalling pathways in the liver of male APP/PS1 transgenic mice

**DOI:** 10.1007/s10522-020-09895-7

**Published:** 2020-08-18

**Authors:** Tímea Téglás, Dóra Ábrahám, Mátyás Jókai, Saki Kondo, Rezieh Mohammadi, János Fehér, Dóra Szabó, Marta Wilhelm, Zsolt Radák

**Affiliations:** 1 Research Center for Molecular Exercise Science, University of Physical Education, Alkotas str. 44, Budapest, 1123 Hungary; 2 Department of Life Sciences, Graduate School of Arts and Sciences, The University of Tokyo, Tokyo, 113-8654 Japan; 3 Ophthalmology Unit, NESMOS Department, Sant’Andrea Hospital, Faculty of Medicine and Psychology, “Sapienza” University of Rome, Rome, Italy; 4 Semmelweis University, Budapest, Hungary; 5 Institute of Sport Sciences and Physical Education, Faculty of Science, University of Pécs, Pecs, 2020 Hungary; 6 Faculty of Sport Sciences, Waseda University, Saitama, 359-1192 Japan

**Keywords:** Alzheimer’s disease, Microbiome, Liver, Probiotics, Exercise, Metabolism

## Abstract

It has been demonstrated that physical exercise and probiotic supplementation delay the progress of Alzheimer’s Disease (AD) in male APP/PS1^TG^ mice. However, it has also been suggested that both exercise and AD have systemic effects. We have studied the effects of exercise training and probiotic treatment on microbiome and biochemical signalling proteins in the liver. The results suggest that liver is under oxidative stress, since SOD2 levels of APP/PS1 mice were decreased when compared to a wild type of mice. Exercise training prevented this decrease. We did not find significant changes in COX4, SIRT3, PGC-1a or GLUT4 levels, while the changes in pAMPK/AMPK, pmTOR/mTOR, pS6/S6 and NRF2 levels were randomly modulated. The data suggest that exercise and probiotics-induced changes in microbiome do not strongly affect mitochondrial density or protein synthesis-related AMPK/mTOR/S6 pathways in the liver of these animals.

## Introduction

The microbiome of the human gastrointestinal tract is the largest reservoir of microbes in the human body. The bacterial density of the human gut is the highest in any known microbial ecosystem (Kim et al. 2013; Hill et al. 2014). The microbiota of the gut is crucial to the breakdown of dietary nutrients, regulation of intestinal and systemic immune responses, and production of small molecules critical for intestinal metabolism, as well as the generation of several gases that can modulate cellular function (Singhal and Shah 2020). Due to the complex function of the gut microbiome, the diversity of microbes is defined by the number and abundance of the distribution of distinct types of organisms (Huttenhower et al. 2012).

The microbiota-gut-brain axis is the most popular hypothesis, which includes a bidirectional communication system, that is connected via neural, immune, endocrine, and metabolic pathways (Cryan and O’Mahony 2011; Collins et al. 2012; Szablewski 2018). Accumulated evidence also supports a possible connection between the gut microbiota and neurodegenerative disorders (Bell et al. 2019), including dementia and Alzheimer’s Disease (AD) in humans (Xu and Wang 2016; Hu et al. 2016) and in rodents (Zhang et al. 2017; Bäuerl et al. 2018). The changes in the microbiome are linked to defects in synaptogenesis, and cognitive impairment, including AD (Aziz et al. 2013; Brenner 2013; Saulnier et al. 2013; Hornig 2013; Mitew et al. 2013).

AD is a complex age-related disorder, characterized by a progressive cognitive decline. A limited number of studies have attempted to address the role of gut microbiota in AD. The colonization and modulation of gut microbiota impact brain development and subsequently, adult behaviour (Heijtz et al. 2011; Mancuso and Santangelo 2018) and this connection will probably aid in AD prevention and treatment (Hu et al. 2016). In humans, Vogt et al. (2017) have described previously the correlation between levels of differentially abundant genera and cerebrospinal fluid (CSF) biomarkers of AD.

Recently, we have shown that probiotic supplementation alone, or with exercise training, or exercise training alone, significantly changes the composition of the microbiome and reduces the levels of amyloid beta in the brain of APP/PS1 transgenic mice (Abraham et al. 2019). It is known that probiotic supplementation also beneficially affects liver in patients suffering from non-alcoholic fatty liver diseases (Liu et al. 2019).

It has been shown that animals having APP/PS1 genetic manipulation demonstrate impaired metabolism in kidney, liver, spleen, and thymus (González-Domínguez et al. 2015a). Indeed, it appears that AD is a systemic disease (González-Domínguez et al. 2015b; Wang et al. 2017). Regular exercise also has systemic effects (Radak et al. 2008), which include powerful beneficial effects on liver (Ghiasi et al. 2019; Aamann et al. 2019; Sato et al. 2019; Zhang et al. 2020). Moreover, evidence suggests that exercise reduces the incidence of AD (Radak et al. 2010; Abe 2012). It has further been shown that short-term physical exercise enhances neurogenesis in the amyloid beta-induced AD model (Kim et al. 2014). Voluntary exercise can decrease amyloid load in theTgCRND8 transgenic mice model (Adlard 2005). Due to the systemic effects of exercise, a number of research studies have found that exercise alters the microbiome of the gut (Allen et al. 2018; Sohail et al. 2019; Grosicki et al. 2019; Mahizir et al. 2020; Gubert et al. 2020; Greenhill 2020).

It is important to know whether exercise and probiotic treatment-induced changes in the microbiome could cause alterations in liver metabolism, mitochondrial content, or protein synthesis. Therefore, we have investigated the effects of physical exercise and probiotic treatment on liver metabolism and the pathophysiology of APP/PS1 transgenic mice, by assessing nuclear factor erythroid 2-related factor 2 (NRF-2), 5′ AMP-activated protein kinase (AMPK), and mammalian target of rapamycin- (mTOR), associated pathways.

## Materials and methods

### Experimental animals

Twenty-four (24), 3-month old, male APP/PS1 transgenic mice (B6C3-Tg (APPswe, PSEN1dE9) 85Dbo/Mmjax; APP/PS1^TG^) and six (6) wild type controls were used in this study. We have previously demonstrated that cognitive performance is decreased in the Morris water maze and Y-maze tests in APP/PS1^TG^ mice (Abraham et al. 2019). Rodents were randomly divided into control (APP/PS1^TG^-C), exercised (APP/PS1^TG^-E), probiotic treated (APP/PS1^TG^-P), and combined (exercise and probiotic treatment, APP/PS1^TG^-E-P) groups (n = 6, in each group). The wild type animals were from the same colony and were used as the absolute control group (Wt) (n = 6). All experimental procedures, which were carried out on the animals, had been approved by the Semmelweis University Ethics Committee (No: PEI/2015–6/2014).

### Experimental design

The experimental groups (E, E-P) were subjected to exercise of interval running on a rodent treadmill for 20 weeks, with the aim of decreasing the progress of AD development and functional impairment. Training was performed four times per week, for 60 min. The training sessions lasted ten cycles, each cycle consisting of four minutes at high intensity (20 m/min) and two minutes low intensity (10 m/min). Animals from the control, probiotic treated, and the wild type control groups were placed on the treadmill for the same time period as the trained animals, without receiving any exercise training.

The probiotic treated mice were supplemented by Framelin, which contains probiotics *Bifidobacterium longum* and *Lactobacillus acidophilus* lysates, vitamins A and D, and omega 3 fatty acids in cod liver oil, as well as vitamins B1, B3, B6, B9, B12. The supplementation of Framelin was carried out five times per week (120 mg/day) for 20 weeks, along with rodent chow. We monitored daily the food uptake of all mice and found that probiotic treatment did not alter the amount of food and water intake of the animals.

After the 20 week treatment period, animals were anesthetized with one dose (0.1 ml/10 g bw) of ketamine via intraperitoneal injection (Richter, 100 mg/ml), and a xylazine (Produlab Pharma, 20 mg/ml) cocktail, and transcardially perfused with heparinized, ice-cold physiological saline. The liver was quickly removed and placed on a frozen iced-cooled glass plate. Fecal samples were collected for microbiome analyses the day before the end of the experiment. The samples were stored at − 80 °C until processing.

14073936–3729-4475-8958-1ad1cf3f27f0

### Western blot analysis

The liver of each animal was homogenized in ice and lysed in a lysis buffer containing 137 mM NaCl, 20 mM Tris–HCl pH 8.0, 1% Nonidet P-40, 10% glycerol, and tablets of protease and phosphatase inhibitors. Lysates were centrifuged for 15 min at 14.000 g at 4 °C. Protein concentration was measured using the Bradford assay (Bradford 1976). Proteins were separated on 8–15% (v/v) SDS-PAGE (sodium dodecyl sulphate–polyacrylamide) gels at room temperature (RT) and transferred onto PVDF membrane (pore size: 0.2 and 0.4 µm) at 4 °C. The nonspecific binding of immune-proteins was blocked with 5% BSA (bovine serum albumin) dissolved in Tris-buffered saline Tween 20 (TBS-T) for 1 h (h) at RT. After blocking, the membranes were incubated with primary GAPDH (mouse, 1:40,000; Sigma-Aldrich), SIRT1 mouse (1:1000, Abcam), SIRT3 rabbit (1:10,000, ProteinTech), rabbit GLUT4 (1:500, Santa Cruz), rabbit SOD2 (1:3000, Invitrogen), goat COX4 (1:2000, Abcam), rabbit PGC1-alpha (1:5000, Novus Biologicals), rabbit S6 Ribosomal Protein (1:5000, Cell Signaling), rabbit Phospho-S6 Ribosomal Protein (1:5000, Ser235/236, Cell Signaling), rabbit AMPK (1:25,000, Cell Signaling), rabbit pAMPK (1:1500, Cell Signaling), rabbit AKT (1:3000, Cell Signaling, rabbit pAKT (1:2000, Cell Signaling), rabbit pmTOR (1:1500, Cell Signaling), rabbit mTOR (1:1000, Cell Signaling) or rabbit NRF-2 (1:1000, Abcam) antibodies in TBS-T containing 5% BSA, overnight at 4 °C. After overnight incubation the membranes were rinsed in TBS-T attended by one hour of incubation with HRP-conjugated secondary antibodies at RT. The secondary antibodies were: anti-rabbit, anti-mouse and anti-goat IgG in TBS-T containing 1% BSA (1:10,000; Jackson Immunoresearch). Between incubation times, the membranes were washed repeatedly (3 × 10 min) and after the last washing session, incubated with an enhanced chemiluminescent reagent (ECL Star Enhanced Chemiluminescent Substrate; Euroclone) for one minute. The protein bands were visualized on X-ray film. Bands were quantified by ImageJ 1.52 software, and total protein normalization was used for the analysis.

### Microbiome analysis

Microbiome was analyzed by 16S ribosomal rDNA amplicon sequencing and bioinformatics as described previously (Abraham et al. 2019).

### Statistical analyses

The Statistica 13.2 program was used for all statistical analyses. The Shapiro–Wilk W-test was used to determine normality. For the parametric variables, three-way ANOVA was used (with the three- degree factorial), followed by the Tukey’s post hoc test. The correlation between two variables was measured using the Pearson coefficient. Statistical significance was established at P < 0.05. Means ± SEMs are shown in the figures.

## Results

### The effects of exercise and probiotics treatment on liver

**Fig. 1:**
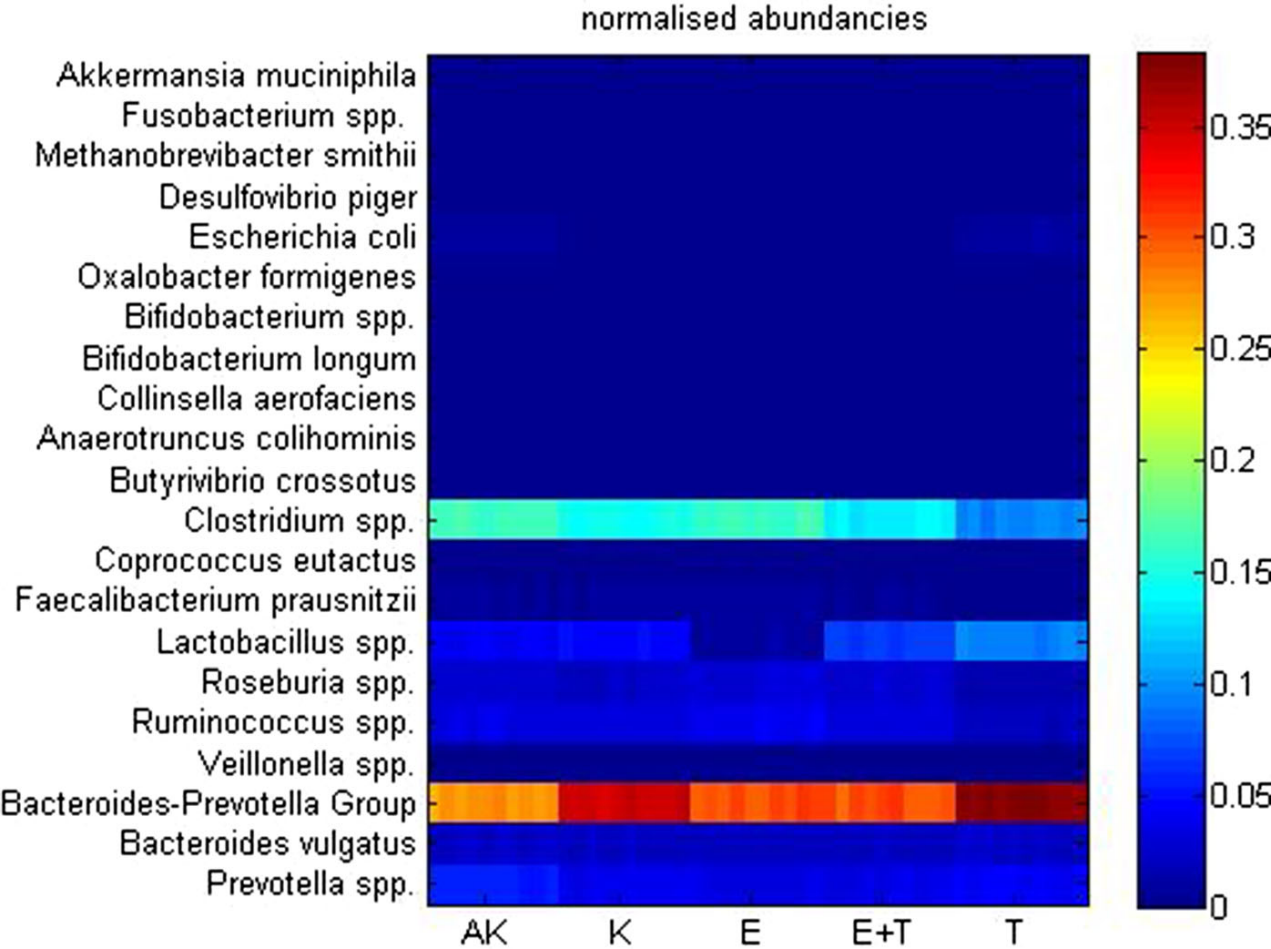
The effects of exercise and probiotic treatments on the gut microbiome of wild and APP/PS1 transgenic mice. The figure shows the bacterial content of wild type (Wt), (APP/PS1^TG^-C), exercise trained (APP/PS1^TG^-E), probiotic treated (APP/PS1^TG^-P), and combined (exercise trained and probiotic treatment, APP/PS1^TG^-E-P) groups (n = 6)

Figure 1 shows the differences in microbiome concentration of wild, and transgenic control, exercise trained, as well as probiotic treated mice. It is clear from these data that the microbiome of APP/PS1^TG^ mice is very different from the wild type, as the effects of both interventions alone or in combination significantly altered the bacterial flora of the microbiome. As we reported earlier, the changes in the bacterium flora in the gut microbiome are associated with brain function and accumulation of amyloid beta proteins (Abraham et al. 2019).

**Fig. 2:**
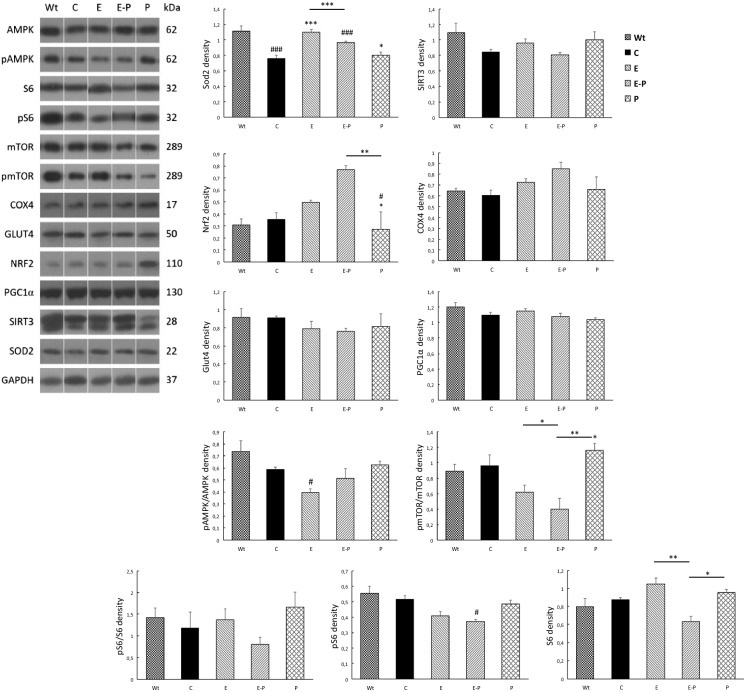
The effects of exercise and probiotic treatments on selected protein contents. Exercise and probiotic treatments randomly altered the selected protein concentrations in the livers of wild and transgenic animals. Wild type (Wt), control (APP/PS1^TG^-C), exercise trained (APP/PS1^TG^-E), probiotic treated (APP/PS1^TG^-P), and combined (exercise trained and probiotic treatment, APP/PS1^TG^-E-P) groups (n = 6). *P < 0.05, **P < 0.01, ***P < 0.001 versus control group, ^#^P < 0.05, ^##^P < 0.01, ^###^P < 0.001 versus Wt group. The lines above the columns indicate the significant differences between the groups (E-P vs. P; E vs. E-P)

In the fecal microbiome *Firmicutes* and *Bacteroidetes* are the most abundant prokaryotic species. Our results revealed that the ratio of the *Firmicutes/Bacteroides* was the lowest in the APP/SP1-P group and the highest in the Wt group without APP/PS1 overexpression. Indeed, in the Wt control group we found a decreased ratio of *Firmicutes* to *Bacteroides species,* compared to the APP/PS1 groups. The level of *L. johnsonii* positively correlated with beta amyloid content and area (p < 0.05) in the hippocampus (Abraham et al. 2019). However, a significant relationship was not detected between the level of bacterial species in the microbiome and the measured protein levels in the liver. Therefore, it is suggested that the impact of these interventions on the liver appears to be less pronounced than found in the brain. Exercise training of probiotic supplementation did not change the mitochondrial content in the liver, judged from the levels of COX4 and PGC-1a proteins (Fig. 2). However, the antioxidant capacity could be affected. The changes of two important mitochondrial proteins, SOD2 and SIRT3, are very similar (Fig. 2), however, significant differences were only observed in SOD2 levels. APP/PS1^TG^ mice have suppressed levels of Mn-SOD compared to wild mice, suggesting elevated sensitivity to oxidative stress. Exercise training and probiotic treatment increased SOD2 protein levels, but the sum effects of these treatments neutralized each other. NRF-2, which is one of the regulators of antioxidant defence, showed a powerful increase in the probiotic supplemented exercising group, while probiotic treatment itself resulted in comparable levels of NRF-2 protein as found for the control group (Fig. 2).

The phosphorylation of AMPK was decreased in the liver of the exercised group, and the pAMPK/AMPK ratio was lowest in this group (Fig. 2). Theoretically, the phosphorylation of AMPK can curb the activity of mTOR. However, in this study we found no difference in the levels of phospho mTOR/mTOR for the wild and control APP/PS1TG mice (Fig. 2) and the pattern of pAMPK/AMPK and phospho mTOR/mTOR showed no strong relationship. The ratio of pmTOR/mTOR tended to decrease in the liver of the exercise trained mice, but probiotic supplementation alone resulted in significant increases in the ratio, when compared to the exercise groups. Surprisingly, the phospho mTOR/mTOR ratio in the liver of combination treated animals, showed similar values to the control animals. In addition, the ratio of phosphorylated and total ribosomal protein S6 kinase was statistically similar in all groups and the levels of GLUT4 in the liver were not altered by the treatments. Correlated data obtained from the microbiome analyses and the immunoblot results for the liver, did not demonstrate any significant relationships.

## Discussion

Alzheimer’s Disease has a powerful impact on brain function. However, data suggest that AD also has systemic effects on the body. It is known, from AD models in rodents, that AD impairs the function of peripheral organs such as liver, kidney, and small intestine (González-Domínguez et al. 2015b; Pan et al. 2019). Metabolomic results from APP/PS1 mouse models revealed that the liver was the organ first affected during the progression of amyloid pathology, demonstrating impaired energy metabolism, amino acid metabolism, nucleic acid metabolism, as well as ketone and fatty acid metabolism (Zheng et al. 2019). In addition the systemic effects of physical exercise have also been well demonstrated (Radak et al. 2008, 2019, 2020). In the present study, we examined the effects of regular exercise, and probiotic treatment on the liver.

Liver experiences a significant drop in blood flow during acute exercise. Because of the central role of the liver in lipid, protein, and carbohydrate metabolism, the regular exercise-mediated adaptation of this organ is of particular importance (Radak et al. 2020).One of the most interesting findings of this study was that the combined effects of exercise training and probiotic supplementation significantly increased the levels of NRF-2 in the liver. NRF-2 is one of the key players of the cellular defense mechanisms against oxidative stress via the activation of antioxidant response element (ARE) genes, including SOD (Magesh et al. 2012). However, it is important to note, that the increase in NRF2 protein levels does not necessarily mean an increase of the activation of ARE, since we do not have information on keap1 expression and translocation of NRF2 (Ma and He 2012).

Besides the role of NRF-2 in the antioxidant system, it is also an important regulator of glucose/glycogen metabolism. Increased levels of NRF-2 in the liver are associated with increased glycogen content, but decreased content in skeletal muscle (Uruno et al. 2016). In the exercise plus probiotics treated group we found enhanced levels of NRF-2, which could indicate that the joint effects of these treatments increased the glycogen stores in the liver, which could be part of an adaptive process to training. However, the levels of GLUT4 remained unchanged. The increased protein content of SOD2 could easily be the NRF-2 mediated induction of ARE. It is important to note, that the SOD2 levels in APP/PS1 transgenic mice were significantly lower than those of the wild types, suggesting a mitochondrial oxidative stress. Indeed, it has been shown that APP/PS1 transgenic animals suffer from impaired mitochondrial transport (Völgyi et al. 2018), increased mitochondrial ROS generation, increased 8-oxodG content, and SOD2 levels in the hippocampus (Bo et al. 2014). Our data suggest, that in this AD model the mitochondrial network, not only in the brain, but also in the liver, is under oxidative stress. Moreover, it is important to note that NRF-2 also has an anti-inflammatory role since it can suppress nuclear factor (NF)κB (Abdelsalam and Safar 2015). It is also known that exercise can curb the age-associated increase in NF-kB activity in rodents (Radák et al. 2004). Our probiotic treatment, Framelin, contained omega 3, B12 vitamin and lactobacillus, the levels of which correlated with lifespan on a caloric restricted model, with reduced inflammation (Zhang et al. 2013). Therefore, the combined effects of exercise and Framelin treatment could suppress the inflammatory process associated with this AD model (Duyckaerts et al. 2007).

The decreased levels of pAMPK/AMPK ratio in the exercised group suggest enhanced gluconeogenesis, which promotes generation of glucose from various sources such as tryglycerides, proteins, and lactate. Decreased pAMPK/AMPK levels and an increased pmTOR/mTOR ratio, could mean increased levels of apoptosis in the liver (Chen et al. 2019). However, in our case, the pmTOR/mTOR ratio in the exercise trained transgenic animals was decreased, albeit not significantly, compared to the control group. This could be an interesting observation, since it has been suggested that inhibition of the mTOR pathway, by nutrient, could lead to an increased life-span (Johnson et al. 2013). On the other hand, the pmTOR/mTOR ratio of the probiotic treated transgenic animals was significantly higher than that of the exercise trained and probiotic treated groups, suggesting that the given probiotic treatment might have pro-aging effects on the liver. This is interesting, since the probiotics we used, Framelin, contained *Bifidobacterium longum*, *Lactobacillus acidophilus* lysates, besides B vitamins and omega 3 fatty acids, which could improve the antioxidant defense (Guo et al. 2017; Liakopoulos et al. 2019), while the alteration of the pmTOR/mTOR pathway, could stimulate pro-aging cellular signaling.

The ribosomal protein S6 kinase is down-stream in the mTOR signaling pathway, which encodes a number of important kinases in the induction of protein synthesis (Tavares et al. 2015). In the present study we could not detect a significant alteration of the pS6/S6 ratio. However, the combined effects of exercise and probiotics did tend to suppress the ratio (p < 0.854).

Overall, the obtained results suggest that liver is under oxidative stress in APP/PS1 transgenic mice, due to the suppressed antioxidant defense, and exercise training has beneficial effects on the antioxidant system in this Alzheimer’s model. Our data suggest that exercise and probiotics-induced changes in the microbiome do not strongly affect the mitochondrial density, and protein synthesis- related AMPK/mTOR/S6 pathways.

## Abbreviations

AD: Alzheimer’s disease
BSA: Bovine serum albumin
HRP: Horseradish peroxidase
mo.: Months
p-S6: Phosphor-ribosomal protein
PVDF: Polyvinylidene fluoride
rpS6: Ribosomal protein
RT: Room temperature
S6: Ribosomal protein
SDS-PAGE: Sodium dodecyl sulphate–polyacrylamide gel electrophoresis
TBS-T: Tris buffered saline with Tween 20

## References

[CR1] Aamann L, Tandon P, Bémeur C (2019). Role of exercise in the management of hepatic encephalopathy: experience from animal and human studies. J Clin Exp Hepatol.

[CR2] Abdelsalam RM, Safar MM (2015). Neuroprotective effects of vildagliptin in rat rotenone Parkinson’s disease model: role of RAGE-NFκB and Nrf2-antioxidant signaling pathways. J Neurochem.

[CR3] Abe K (2012). Total daily physical activity and the risk of ad and cognitive decline in older adults. Neurology.

[CR4] Abraham D, Feher J, Scuderi GL (2019). Exercise and probiotics attenuate the development of Alzheimer’s disease in transgenic mice: role of microbiome. Exp Gerontol.

[CR5] Adlard PA (2005). Voluntary exercise decreases amyloid load in a transgenic model of Alzheimer’s disease. J Neurosci.

[CR6] Allen JM, Mailing LJ, Niemiro GM (2018). Exercise alters gut microbiota composition and function in lean and obese humans. Med Sci Sports Exerc.

[CR7] Aziz Q, Doré J, Emmanuel A (2013). Gut microbiota and gastrointestinal health: current concepts and future directions. Neurogastroenterol Motil.

[CR8] Bäuerl C, Collado MC, Diaz Cuevas A (2018). Shifts in gut microbiota composition in an APP/PSS1 transgenic mouse model of Alzheimer’s disease during lifespan. Lett Appl Microbiol.

[CR9] Bell JS, Spencer JI, Yates RL (2019). Invited review: from nose to gut – the role of the microbiome in neurological disease. Neuropathol Appl Neurobiol.

[CR10] Bo H, Kang W, Jiang N (2014). Exercise-induced neuroprotection of hippocampus in APP/PS1 transgenic mice via upregulation of mitochondrial 8-oxoguanine DNA glycosylase. Oxid Med Cell Longev.

[CR11] Bradford MM (1976). A rapid and sensitive method for the quantitation of microgram quantities of protein utilizing the principle of protein-dye binding. Anal Biochem.

[CR12] Brenner SR (2013). Blue-green algae or cyanobacteria in the intestinal micro-flora may produce neurotoxins such as Beta-N-Methylamino-l-Alanine (BMAA) which may be related to development of amyotrophic lateral sclerosis, Alzheimer’s disease and Parkinson-Dementia-Complex in. Med Hypotheses.

[CR13] Chen X, Li C, Chen Y (2019). Aflatoxin B1 impairs leydig cells through inhibiting AMPK/mTOR-mediated autophagy flux pathway. Chemosphere.

[CR14] Collins SM, Surette M, Bercik P (2012). The interplay between the intestinal microbiota and the brain. Nat Rev Microbiol.

[CR15] Cryan JF, O’Mahony SM (2011). The microbiome-gut-brain axis: from bowel to behavior. Neurogastroenterol Motil.

[CR16] Duyckaerts C, Potier M-C, Delatour B (2007). Alzheimer disease models and human neuropathology: similarities and differences. Acta Neuropathol.

[CR17] Ghiasi R, Naderi R, Sheervalilou R, Alipour MR (2019). Swimming training by affecting the pancreatic Sirtuin1 (SIRT1) and oxidative stress, improves insulin sensitivity in diabetic male rats. Horm Mol Biol Clin Investig.

[CR18] González-Domínguez R, García-Barrera T, Vitorica J, Gómez-Ariza JL (2015). High throughput multiorgan metabolomics in the APP/PS1 mouse model of Alzheimer’s disease. Electrophoresis.

[CR19] González-Domínguez R, García-Barrera T, Vitorica J, Gómez-Ariza JL (2015). Metabolomic investigation of systemic manifestations associated with Alzheimer’s disease in the APP/PS1 transgenic mouse model. Mol Biosyst.

[CR20] Greenhill C (2020). Gut microbiome influences exercise response. Nat Rev Endocrinol.

[CR21] Grosicki GJ, Durk RP, Bagley JR (2019). Rapid gut microbiome changes in a world-class ultramarathon runner. Physiol Rep.

[CR22] Gubert C, Kong G, Renoir T, Hannan AJ (2020). Exercise, diet and stress as modulators of gut microbiota: implications for neurodegenerative diseases. Neurobiol Dis.

[CR23] Guo Q, Li S, Xie Y (2017). The NAD+-dependent deacetylase, Bifidobacterium longum Sir2 in response to oxidative stress by deacetylating FOXO3a and SigH (σH) in Bifidobacterium longum and HEK293T cells respectively. Free Radic Biol Med.

[CR24] Heijtz RD, Wang S, Anuar F (2011). Normal gut microbiota modulates brain development and behavior. Proc Natl Acad Sci USA.

[CR25] Hill JM, Bhattacharjee S, Pogue AI, Lukiw WJ (2014). The gastrointestinal tract microbiome and potential link to Alzheimer’s disease. Front Neurol.

[CR26] Hornig M (2013). The role of microbes and autoimmunity in the pathogenesis of neuropsychiatric illness. Curr Opin Rheumatol.

[CR27] Hu X, Wang T, Jin F (2016). Alzheimer’s disease and gut microbiota. Sci China Life Sci.

[CR28] Huttenhower C, Gevers D, Knight R (2012). Structure, function and diversity of the healthy human microbiome. Nature.

[CR29] Johnson SC, Rabinovitch PS, Kaeberlein M (2013). MTOR is a key modulator of ageing and age-related disease. Nature.

[CR30] Kim B-K, Shin M-S, Kim C-J (2014). Treadmill exercise improves short-term memory by enhancing neurogenesis in amyloid beta-induced Alzheimer disease rats. J Exerc Rehabil.

[CR31] Kim B-S, Jeon Y-S, Chun J (2013). Current status and future promise of the human microbiome. Pediatr Gastroenterol Hepatol Nutr.

[CR32] Liakopoulos V, Roumeliotis S, Bozikas A (2019). Antioxidant supplementation in renal replacement therapy patients: is there evidence?. Oxid Med Cell Longev.

[CR33] Liu L, Li P, Liu Y, Zhang Y (2019). Efficacy of probiotics and synbiotics in patients with nonalcoholic fatty liver disease: a meta-analysis. Dig Dis Sci.

[CR34] Ma Q, He X (2012). Molecular basis of electrophilic and oxidative defense: promises and perils of Nrf2. Pharmacol Rev.

[CR35] Magesh S, Chen Y, Hu L (2012). Small molecule modulators of keap1-Nrf2-ARE pathway as potential preventive and therapeutic agents. Med Res Rev.

[CR36] Mahizir D, Briffa JF, Wood JL (2020). Exercise improves metabolic function and alters the microbiome in rats with gestational diabetes. FASEB J.

[CR37] Mancuso C, Santangelo R (2018). Alzheimer’s disease and gut microbiota modifications: the long way between preclinical studies and clinical evidence. Pharmacol Res.

[CR38] Mitew S, Kirkcaldie MTK, Dickson TC, Vickers JC (2013). Altered synapses and gliotransmission in alzheimer’s disease and AD model mice. Neurobiol Aging.

[CR39] Pan Y, Omori K, Ali I (2019). Increased expression of renal drug transporters in a mouse model of familial Alzheimer’s disease. J Pharm Sci.

[CR40] Radak Z, Chung HY, Goto S (2008). Systemic adaptation to oxidative challenge induced by regular exercise. Free Radic Biol Med.

[CR41] Radák Z, Chung HY, Naito H (2004). Age-associated increase in oxidative stress and nuclear factor kappaB activation are attenuated in rat liver by regular exercise. FASEB J.

[CR42] Radak Z, Hart N, Sarga L (2010). Exercise plays a preventive role against Alzheimer’s disease. J Alzheimer’s Dis.

[CR43] Radak Z, Suzuki K, Posa A (2020). The systemic role of SIRT1 in exercise mediated adaptation. Redox Biol.

[CR44] Radak Z, Torma F, Berkes I (2019). Exercise effects on physiological function during aging. Free Radic Biol Med.

[CR45] Sato Y, Qiu J, Miura T (2019). Effects of long-term exercise on liver cyst in polycystic liver disease model rats. Med Sci Sport Exerc.

[CR46] Saulnier DM, Ringel Y, Heyman MB (2013). The intestinal microbiome, probiotics and prebiotics in neurogastroenterology. Gut Microbes.

[CR47] Singhal R, Shah YM (2020). Oxygen battle in the gut: hypoxia and hypoxia-inducible factors in metabolic and inflammatory responses in the intestine. J Biol Chem.

[CR48] Sohail MU, Yassine HM, Sohail A, Al Thani AA (2019). Impact of physical exercise on gut microbiome, inflammation, and the pathobiology of metabolic disorders. Rev Diabet Stud.

[CR49] Szablewski L (2018). Human gut microbiota in health and Alzheimer’s disease. J Alzheimer’s Dis.

[CR50] Tavares MR, Pavan ICB, Amaral CL (2015). The S6K protein family in health and disease. Life Sci.

[CR51] Uruno A, Yagishita Y, Katsuoka F (2016). Nrf2-mediated regulation of skeletal muscle glycogen metabolism. Mol Cell Biol.

[CR52] Vogt NM, Kerby RL, Dill-McFarland KA (2017). Gut microbiome alterations in Alzheimer’s disease. Sci Rep.

[CR53] Völgyi K, Badics K, Sialana FJ (2018). Early presymptomatic changes in the proteome of mitochondria-associated membrane in the APP/PS1 mouse model of Alzheimer’s disease. Mol Neurobiol.

[CR54] Wang J, Gu BJ, Masters CL, Wang Y-J (2017). A systemic view of Alzheimer disease: insights from amyloid-β metabolism beyond the brain. Nat Rev Neurol.

[CR55] Xu R, Wang Q (2016). Towards understanding brain-gut-microbiome connections in Alzheimer’s disease. BMC Syst Biol.

[CR56] Zhang C, Li S, Yang L (2013). Structural modulation of gut microbiota in life-long calorie-restricted mice. Nat Commun.

[CR57] Zhang L, Wang Y, Xiayu X (2017). Altered gut microbiota in a mouse model of Alzheimer’s disease. J Alzheimer’s Dis.

[CR58] Zhang X, Cao L, Ji B (2020). Endurance training but not high-intensity interval training reduces liver carcinogenesis in mice with hepatocellular carcinogen diethylnitrosamine. Exp Gerontol.

[CR59] Zheng H, Cai A, Shu Q (2019). Tissue-specific metabolomics analysis identifies the liver as a major organ of metabolic disorders in amyloid precursor protein/presenilin 1 mice of Alzheimer’s disease. J Proteome Res.

